# Soil inhabiting bacto-helmith complex in insect pest management: Current research and future challenges

**DOI:** 10.1016/j.heliyon.2024.e36365

**Published:** 2024-08-15

**Authors:** Preety Tomar, Neelam Thakur, Sangram Singh, Sanjeev Kumar, Sarvesh Rustagi, Ashutosh Kumar Rai, Sheikh Shreaz, Neelam Yadav, Pankaj Kumar Rai, Ajar Nath Yadav

**Affiliations:** a Department of Zoology, Akal College of Basic Sciences, Eternal University, Baru Sahib, Sirmour-173101, Himachal Pradesh, India; b Department of Biochemistry, Dr. Ram Manohar Lohia Avadh University, Ayodhya, Uttar Pradesh, India; c Faculty of Agricultural Sciences, GLA University, Mathura, Uttar Pradesh, India; d Depratment of Food Technology, School of Applied and Life Sciences, Uttaranchal University, Dehradun, Uttarakhand, India; e Department of Biochemistry, College of Medicine, Imam Abdulrahman Bin Faisal University, Dammam, Kingdom of Saudi Arabia; f Desert Agriculture and Ecosystem Department, Environment and Life Sciences Research Center, Kuwait Institute for Scientific Research, P. O. Box 24885, 13109, Safat, Kuwait; g Centre of Research Impact and Outcome, Chitkara University, Rajpura-140401, Punjab, India; h Chitkara Center for Research and Development, Chitkara University, Himachal Pradesh-174103, India; i Department of Biotechnology, Invertis University, Bareilly, Uttar Pradesh, India; j Department of Genetics, Plant Breeding and Biotechnology, Dr. Khem Singh Gill Akal College of Agriculture, Eternal University, Baru Sahib, Sirmaur-173101, Himachal Pradesh, India

**Keywords:** Biocontrol, Entomopathogens, Formulations, Infectivity, Pesticides

## Abstract

Pesticides have health consequences for humans, living organisms, and ecosystems. Research on biological management, with a primary focus on entomopathogens, has been accelerated by the rise in issues such as pesticide residue, soil degradation, and pest resistance. Entomopathogenic nematodes (EPNs) are among the most frequently used and commercialised biopesticides. However, they are restricted in their infectivity, persistence, storage, and cost of production. The nematodes, along with their endosymbiotic bacteria, combine to form a nemato-bacterial complex. This complex is responsible for causing mortality in insect pests due to the production of insecticidal compounds. The adaptation of EPNs is an eco-friendly method, economical, and safer for the environment as well as non-target organisms. Moreover, it's a better alternative to synthetic chemical pesticides, as it can be helpful in overcoming pest resistance and resurgence issues. Application of nematode juveniles is a cost-effective method, but the necessity of refrigeration and transportation may enhance their cost. This review emphasised the diversity of entomopathogenic nematodes and their endosymbiotic bacteria, the exploration of the biocontrol potential of insect pests by under-utilisation of nematodes, the development of nematode-based formulations, and the discussion of critical issues and required research in the future.

## Highlights

• Portrayal of the distribution of entomopathogenic nematodes and their bacteria.
• Biocontrol potential of entomopathogenic nematodes against insect pests.
• Description of the nematode-based bioformulations available on the market.

## 1 Introduction

Crop productivity for human consumption is a menace because of the prevalence and outbreak of insect pests in the agricultural industry. According to Food and Agricultural Organisation (FAO) statistics, an average 40 % loss in crop yield is due to the attack of insect pests, which account for $290 billion of the global economy [1]. The losses in crop productivity caused by these insect pests can be extensive and reduced by adapting crop protection measures [2]. Although several chemical-based insecticides and pesticides have been employed to manage the insect pest's populations, These pesticides enhanced economic potential, increased food productivity, and killed insects [3].

Pesticides possess a broad spectrum of compounds, such as antifungal, insecticidal, rodenticides, herbicides, nematicides, and mollusc-killing compounds, along with some plant growth regulators. Pesticides have grievous health consequences for humans, living organisms, and ecosystems. Large numbers of pest insects have developed resistance to the insecticides [4]. Immense evidence is available that indicates that synthetic chemicals possess potential hazards to humans and non-target organisms and cause other ill effects on the environment [5]. So an alternative economic and eco-friendly method was required to overcome the hazards of insecticides and pesticides. Applications of biocontrol agents such as entomopathogenic nematodes (EPNs) are the best alternative method to conquer all these problems.

Entomopathogenic nematodes, or insect-killing worms, are free-living organisms ubiquitous in ecosystems. These worms are mainly known to parasitize insects. Various kinds of interactions were seen among nematodes and insects, including phoretic, symbiotic, commensalistic, facultative, and obligatory interactions [6]. Soil offered admirable circumstances for EPNs survival [7]. The order Rhabditida has two main genus of EPNs, namely *Steinernema* under the family Steinernematidae and *Heterorhabditis* under the family Heterorhabditidae, with a total of 117 described species of EPNs. A total of 100 species in the genus *Steinernema* and 17 species from the genus *Heterorhabditis* have been reported worldwide [8]. The first EPN, *Aplectana kraussei,* was identified by Steiner in 1923. Genus *Heterorhabditis* was described by Poinar Jr [9]. In the 1980s, the biocontrol potential of EPNs was realised by scientists, which became the turning point of nematode-related research [10].

A complex way in which nematodes behave has been observed due to stimuli (chemical or physical) [11]. Five such habitual approaches were exhibited by nematodes: nictation, ambushing, jumping, cruising, and coalescences of ambush with cruising [12]. These behavioural approaches help the nematodes search for their host. Cruising behaviour is shown by highly mobile nematodes (*Heteriorhabditis bacteriophora* Poinar) that locate their host via their response to the chemical cues in the soil [13]. Ambusher behavioural approach exhibited by ambulatory nematodes that remains steady and attached to the passing host [12]. The ambusher sometimes lifts itself, standing over its tail and the entire body hanging like a loop. This is a kind of nictation [14]. *Steinernema feltiae* (Filipjev) exhibits a combination of both ambushers and cruisers. They are able to parasitize both immovable and movable hosts. *Steinernema scapterisci* (Nguyen and Smart) showed jumping actions where they attached to the insect body and migrated through phoresis [15].

The EPNs life cycle consists of three developmental stages: eggs, juveniles (04) and adults (male and female, or hermaphrodite female). Most of the life stages of EPNs were found feeding, growing, and reproducing inside the dead host. Only the “dauer juveniles (DJs), also aliased as the third juvenile form (IJ3), rm (IJ3), were found free-living in the environment or soil [16]. This third phase of juveniles (IJs) is regarded as the recovery stage and is actually responsible for invading and parasitizing the host insect [17]. As they perceive the host, the worm exuviates its cuticular lining to reveal the body openings [18]. They puncture the insect body through the natural interstice [19], including several entry routes such as anterior openings (mouth), posterior openings (anus), and the body surface opening (spiracle), along with the cuticular lining [20].

The nematodes showed a mutualistic alliance with the bacterial species that inhabit the alimentary canal of these nematodes. They combine to form a nemato-bacterial complex. The endosymbionts of the genus *Steinernema* contain the genus *Xenorhabdus,* and the nematode *Heterorhabditis* showed a mutual interrelation with the genus *Photorhabdus* [21]. The endosymbiotic bacteria of the genus *Xenorhabdus* and *Photorhabdus* are the members of family enterobacteriaceae under the order Enterobacterales and class Gammaproteobacteria [20]. The bacterial symbionts employed nematodes as a vector to get entry inside insects and devoted some part of their life cycle to the nematodes [22]. They effectively invade and parasitize the larval stages of soil inhabiting insect communities [23]. In the case of *Steinernema* spp., the bacterial cells were harboured in the special intestinal vesicles of the nematode, while in *Heterorhabditis,* the bacterial cells reside in the gut region and can be found scattered in the digestive tract of the nematode [24]. In *Heterorhabditis* spp., endosymbionts were regurgitated, while in *Steinernema* spp., they were expelled through the anal region [25].

The bacterial cells proliferated inside the insect haemocoel and, within 24–48 h, killed its host insect [26]. The bacteria released antimicrobial compounds that have immunosuppressive factors and a plethora that eroded with the host's tissues and created a nutritional medium for the nematode's development and reproduction [27]. In the case of *Heterorhabditis* nematodes hermaphrodites (hermaphroditic), females were produced in the first generation, but in the next generation, males and females (amphimictic) were developed. *Steinernema* species always produced amphimictic generations (males and females) [28]. The adults mate and lay eggs that hatch, and again the juveniles moulted and developed into adults. The cycle persisted for up to three generations until the nutrients were exhausted [29]. Due to an exhausting nutrient supply, the IJ_3_ colonised the endosymbionts inside their body, ripped the empty host cadaver, and quested for the new host [30].

EPNs of the genera *Steinernema* and *Heterorhabditis* have been effectively utilised as bio-agents in numerous insect management programs. One of the reasons for EPN utilisation as a bioagent is that 90 % or more insect pests inhabit soil to complete their life cycle. So they have been efficiently applied against ground-inhabited as well as folivore insects [31]. Due to their virulence, motility, quick and adaptive nature, and good sense of chemoception, EPNs have been effectively utilised in agricultural systems [32]. Additionally, they are eco-friendly, conservative, and free of harm [[33], [34], [35]]. Along with all the idiosyncrasy, the culturing of EPNs *in vivo* and *in vitro* is quite easy [36]. Even the applications of these EPNs are facile and can be showered via knapsack sprayer [37]. They are well-established biocontrol agents (BCA) and have been successfully employed in agricultural insect pest management strategies [38]. The attention paid to the biocontrol potential of EPNs began in the late 1930s. Although EPNs were familiar since the 17th century [39]. Immense studies on EPNs were accomplished in the 19th and 20th centuries (Table 1). This review emphasised the distribution of entomopathogenic nematodes and their endosymbiotic bacteria, the exploration of the biocontrol potential of insect pests by under-utilisation of nematodes, the development of nematode-based formulations, and the discussion of critical issues and required research in the future.

**Table 1:** Global distribution of described species of entomopathogenic nematodes.

Country	Nematode Species	Bait insect/Host organism	Habitat	References
**Argentina**	*Steinernema ritteri*	*Galleria mellonella*	Soil	[105]
	*Steinernema rarum*	*G. mellonella*	Soil	[106]
**Australia**	*Heterorhabditis bacteriophora*	*Heliothis punctigera*	Soil	[107]
	*Steinernema* spp.	*G. mellonella*	Soil	[44]
**Brazil**	*Heterorhabditis amazonensis*	*G. mellonella*	Soil	[108]
	*Steinernema brazilense*	*G. mellonella*	Soil	[109]
**Benin**	*Steinernema kandii*	*G. mellonella*	Eucalyptus	[110]
**Cameroon**	*Steinernema cameroonense*	*G. mellonella*	Soil	[111]
	*Steinernema nyetense*	*G. mellonella*	Soil	[111]
**Chile**	*Steinernema australe*	*G. mellonella*	Soil	[112]
	*Steinernema unicornum*	*G. mellonella*	Soil	[113]
	*Heterorhabditis atacamensis*	*G. mellonella*	Soil	[114]
**China**	*Steinernema caudatum*	*G. mellonella*	Soil	[115]
	*Steinernema longicaudum*	*G. mellonella*	Soil	[116]
	*Steinernema ceratophorum*	*G. mellonella*	Soil	[117]
	*Steinernema guangdongense*	*G. mellonella*	Eucalypt forest	[118]
	*Steinernema beddingi*	*G. mellonella*	Soil	[119]
	*Steinernema akhursti*	*G. mellonella*	Soil	[119]
	*Steinernema aciari*	*G. mellonella*	Coastal area soil	[120]
	*Steinernema hebeiense*	*G. mellonella*	Sandy soil	[121]
	*Steinernema sichuanense*	*G. mellonella*	Roadside soil	[122]
	*Steinernema leizhouense*	*G. mellonella*	Soil	[123]
	*Steinernema cholashanense*	*G. mellonella*	Soil	[124]
	*Steinernema xueshanense*	*G. mellonella*	Dry meadows soil	[125]
	*Steinernema pui*	*G. mellonella*	Rubber plantations	[126]
	*Steinernema xinbinense*	*G. mellonella*	Soil	[127]
	*Steinernema tielingense*	*G. mellonella*	Shrubs	[127]
	*Steinernema changbaiense*	*G. mellonella*	Soil	[128]
	*Heterorhabditis beicherriana*	*G. mellonella*	Cherry orchard	[129]
**Colombia**	*Steinernema colombiense*	*G. mellonella*	Coffee plantations	[130]
**Costa Rica**	*Steinernema puntauvense*	*G. mellonella*	Soils	[131]
	*Steinernema costaricense*	*G. mellonella*	volcanic soils	[131]
**Cuba**	*Steinernema cubanum*	*G. mellonella*	Citrus plantation soil	[132]
**Czech** **Republic**	*Steinernema poinari*	*G. mellonella*	Sandy soil	[133]
	*Steinernema weiseri*	*G. mellonella*	Fruit orchards and crop fields	[134]
**Czechoslovakia**	*Steinernema carpocapsae*	*Cydia pomonella*	–	[135]
**Denmark**	*Steinernema affine*	*Phyla febrilis*	Preserved specimens	[136]
	*Steinernema feltiae*	*Feltia segetum (Agrotis segetum)*	Preserved specimens	[137]
**Egypt**	*Heterorhabditis taysearae*	*G. mellonella*	*Hibiscus micranthus*	[138]
**Ethiopia**	*Steinernema yirgalemense*	*G. mellonella*	Field soil	[139]
	*Steinernema ethiopiense*	*G. mellonella*	Soil	[140]
**France**	*Steinernema boemarei*	*G. mellonella*	Soil	[141]
**Germany**	*Steinernema kraussei*	*Cephaleia abietis (L.)*	*-*	[142]
	*Steinernema silvaticum*	*G. mellonella*	Woodland soil	[143]
	*Steinernema schliemanni*	*Osmoderma ceremita*	Soil	[144]
**Indonesia**	*Steinernema hermaphroditum*	*G. mellonella*	Different vegetation	[145]
**Iran**	*Steinernema arasbaranense*	*G. mellonella*	Arasbaran forest	[146]
**Italy**	*Steinernema apuliae*	*G. mellonella*	Soil	[147]
	*Steinernema ichnusae*	*G. mellonella*	Sandy soil of coastal region	[148]
	*Steinernema vulcanicum*	*G. mellonella*	Chestnut wood	[149]
**India**	*Heterorhabditis indica*	*Scirpophaga excerptalis*	Sugarcane field soil	[150]
	*Heterorhabditis casmirica*	*G. mellonella*	Agricultural soils	[151]
	*Steinernema shori*	*G. mellonella*	Soil	[152]
	*Steinernema anantnagense*	*G. mellonella*	Agricultural fields soils	[153]
**Ireland**	*Heterorhabditis downesi*	*G. mellonella*	Grassland soil	[154]
**Japan**	*Steinernema kushidai*	*G. mellonella*	Soil from five different climates	[155]
	*Steinernema litorale*	*G. mellonella*	Pine forest of coastal region	[156]
	*Steinernema ashiuense*	*G. mellonella*	Grassland soil	[157]
**Korea**	*Steinernema monticolum*	*Agrotis segetum; Agrotis ipsilon,*	Forest, Chestnut	[158]
**Kenya**	*Steinernema karii*	*G. mellonella*	Soil	[159]
**Mexico**	*Heterorhabditis mexicana*	*G. mellonella*	Soil	[160]
	*Steinernema ralatorei*	*G. mellonella*	Soil	[161]
	*Heterorhabditis zacatecana*	*G. mellonella*	Maize fields	[162]
**Nepal**	*Steinernema lamjungense*	*G. mellonella*	Soil	[163]
	*Steinernema surkhetense*	*G. mellonella*	Soil	[164]
	*Steinernema nepalense*	*G. mellonella*	Soil	[164]
**New Zealand**	*Heterorhabditis zealandica*	*Heteronychus arator*	–	[165]
**Oregon**				
**Pakistan**	*Steinernema pakistanense*	*G. mellonella*	Soil	[166]
	*Steinernema asiaticum*	*G. mellonella*	Soil	[167]
	*Steinernema bifurcatum*	*G. mellonella*	Rose plant	[168]
	*Steinernema balochiense*	*G. mellonella*	*Psidium guajava*	[169]
**Poland**	*Steinernema sandneri*	*G. mellonella*	Soil	[170]
**Puerto Rico**	*Steinernema puertoricense*	*G. mellonella*	Coconut plantations	[171]
**Russia**	*Steinernema arenarium*	–	Preserved specimens	[137]
**Republic of Rwanda**	*Heterorhabditis ruandica*	*G. mellonella*	Cropland soil	[162]
	*Steinernema africanum*	*G. mellonella*	Agricultural soils	[172]
**Spain**	*Steinernema riojaense*	*G. mellonella*	Soil from Grapevine field	[173]
**South Africa**	*Steinernema citrae*	*G. mellonella, T. molitor*	Soil from citrus orchard	[174]
	*Steinernema innovationi*	*G. mellonella*	Grain field	[175]
	*Steinernema beitlechemi*	*G. mellonella*	Grassland soil	[176]
	*Steinernema biddulphi*	*G. mellonella*	Maize field	[177]
	*Steinernema fabii*	*G. mellonella*	Soil from Black wattle plantation	[178]
	*Steinernema jeffreyense*	*G. mellonella*	Guava tree	[179]
	*Steinernema khoisanae*	Whitegrub and black vine weevil	Soil from fruit orchards	[180]
	*Steinernema litchii*	*T. molitor*	Soil from litchi orchards	[181]
	*Steinernema nguyeni*	*G. mellonella*	Soil from *Olea africana* tree	[182]
	*Steinernema sacchari*	*Eldana saccharina, G. mellonella*	Soil from sugarcane field	[183]
	*Steinernema tophus*	*G. mellonella*	Vineyard soil	[184]
	*Steinernema bertusi*	*G. mellonella*	Soil from wattle plantation	[185]
	*Steinernema batswanae*	*G. mellonella*	Sub-tropical woodland area	[186]
	*Heterorhabditis safricana*	*G. mellonella*	Soil from peach orchard	[187]
	*Heterorhabditis noenieputensis*	*G. mellonella*	Soil from fig tree	[188]
	Undescribed spp.	*G. mellonella*	Citrus orchard	[189]
**Serbia**	*Steinernema bicornutum*	*G. mellonella*	Soil	[190]
**Sultanate of Oman**	*Steinernema abbasi*	*G. mellonella*	Sandy soil	[191]
**Taiwan**	*Steinernema taiwanensis*	*Spodoptera litura*	Grassland soil	[192]
**Turkey**	*Steinernema anatoliense*	*G. mellonella*	Grassland soil	[193]
**Tanzania**	*Steinernema pwaniensis*	*G. mellonella*	Loamy sand soil	[194]
**Thailand**	*Steinernema siamkayai*	*G. mellonella*	Soil from sweet tamarind orchard	[195]
	*Steinernema minutum*	*-*	Soil	[196]
**USA**	*Steinernema glaseri*	*Popillia japonica*	Soil	[197]
	*Steinernema riobrave*	*Helicoverpa* zea	Soil from corn field	[198]
	*Steinernema scarabaei*	*Anomala orientalis* *Popillia japonica*	Turfgrass	[199]
	*Steinernema texanum*	*G. mellonella*	Soil from mixed weedy vegetation	[200]
	*Steinernema intermedium*	*G. mellonella*	–	[201]
	*Steinernema* spp.	*G. mellonella*	Soil	[202]
	*Steinernema jollieti*	*G. mellonella*	–	[203]
	*Heterorhabditis georgiana*	*G. mellonella*	Soil from pecan orchard	[204]
	*Steinernema tbilisiensis*	*G. mellonella*	Soil from deciduous forest	[205]
	*Steinernema borjomiense*	*Oryctes nasicornis, G. mellonella*	**-**	[206]
	*Heterorhabditis megidis*	*Popillia japonica*	Soil	[207]
	*Heterorhabditis marelatus*	*G. mellonella*	Soil	[208]
	*Steinernema neocurtillae*	*Neocurtilla hexadactylla*	Field soil	[209]
	*Steinernema diaprepesi*	*Diaprepes abbreviates, G. mellonella*	Field soil	[210]
	*Heterorhabditis floridensis*	*G. mellonella*	Citrus plantations	[211]
	*Steinernema phyllophagae*	*Phyllophaga* sp.	Oak	[212]
	*Steinernema khuongi*	*G. mellonella*	Citrus tree	[213]
	*Steinernema oregonense*	*G. mellonella*	Soil	[214]
**Uruguay**	*Steinernema scapterisci*	*Scapteriscus vicinus*	–	[215]
**Vietnam**	*Steinernema robustispiculum*	*G. mellonella*	Woodland	[216]
	*Steinernema tami*	*G. mellonella*	Cat Tien forest	[217]
	*Steinernema sangi*	*G. mellonella*	Soil	[218]
	*Steinernema thanhi*	*G. mellonella*	beach soil	[219]
	*Steinernema loci*	*G. mellonella*	beach soil	[219]
	*Steinernema sasonense*	*G. mellonella*	Forest soil	[220]
	*Steinernema cumgarense*	*G. mellonella*	Forest soil	[220]
	*Steinernema backanense*	*G. mellonella*	Forest soil	[220]
	*Steinernema eapokense*	*G. mellonella*	Forest soil	[220]
	*Steinernema huense*	*G. mellonella*	Forest soil	[221]
	*Heterorhabditis baujardi*	*G. mellonella*	Forest soil	[222]
**Venezuela**	*Steinernema papillatum*	*G. mellonella*	Grassland	[223]
	*Steinernema goweni*	*G. mellonella*	Fallow field	[224]

## 2 Nemato-bacterial complex

**Fig. 1:**
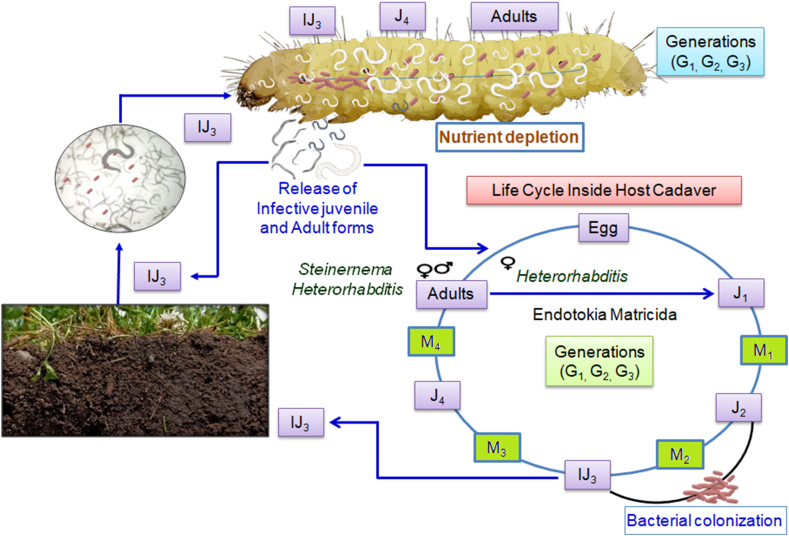
Life cycle of entomopathogenic nematodes; IJ: Infective Juvenile; G: generation; M: Moulting; J: Juvenile form [292].

The nematode-associated endosymbiotic bacteria *Achromobacter nematophilus* were first described by Poinar Jr. and Thomas [40] from infective juveniles of *Steinernema carpocapsae* (Weiser). They illustrated the position of this endosymbiont inside the nematode juvenile using microscopic observations, including a compound, as well as electronic microscopy techniques [41]. He also explained the contribution of this bacterium to the life cycle of the nematode (Fig. 1). He also demonstrated the key role of this bacterium in the host's death. Thomas and Poinar assigned a new genus to this bacterium, which was designated as genus *Xenorhabdus* on the basis of its identifying features [42]. The endosymbiotic bacterium was isolated from *H. bacteriophora* [43], and Thomas and Poinar named it *Xenorhabditis luminescens* because of its fluorescence ability [42]. Later, it was realised that the bacterium possessed different phenotypic and genotypic characters, so the newly isolated bacterium was placed in a separate genus designated as *Photorhabdus* [44].

*Xenorhabdus* bacteria were located inside the special vesicle inside the nematode intestines [45], while *Photorhabdus* bacteria were disseminated in the digestive tract, including the fore and midgut regions of *Heterorhabditis* juveniles [46]. All *Steinernema* showed mutualistic symbiotic associations with the genus *Xenorhabdus,* and *Heterorhabditis* species showed symbiosis with the genus *Photorhabdus* [47]. The symbiosis mechanism between EPNs and their endosymbiotic bacteria was a chief turning point for the utilisation of EPNs as bioagents. Based on their phenotype, these bacteria are Gram-negative, non-spore-forming, facultative anaerobes, and rod-like in appearance [48]. These bacteria are well known for their phenotypic dissimilarities and exhibit a mechanism of phase variation. The bacteria exhibit digenetic forms that show phase variation in their life forms [49]. Phase I was the metabolic phase with primary cells that imparted a red colour, or sometimes brick red colour, to the colony with characteristic bioluminescence properties. The phase II bacterial cells showed glistening, whitish colonies that exhibit non-bioluminescence characters. Earlier, it was reported that *Photorhabdus luminescens* (Thomas and Poinar) shows red-coloured colonies overlaid by the dark blue colour of the media [50]. The colonies of *P. luminescens* were observed to be circular, smooth, glistering, convex, and had white margins with a shiny appearance [51].

Phase-I is denoted as the primary phase variant found inside the infective juvenile stage of the nematode. It is more virulent and binds with specific dyes. It produced a wide range of metabolic compounds [52]. Phase II, or the secondary variant, is produced when resources (low oxygen and osmolarity levels) are exhausted [53] and the organism is less virulent. Phase II cells have low antibiotic production, untraceable bioluminescence, less pigmentation, and lower virulence than phase I cells [54]. Both phases help in the growth, development, and reproduction of the nematodes [55]. Smigielski et al. reported that phase I cells enhanced nematode growth and development during bacterial-nematode interactions [53]. Eckstein and Heermann also described the phenotypic switching among the primary and secondary cells of *P. luminescens* [56]. Tu et al. reported the two phases in the life cycle of *Photorhabdus* species that exhibit different secondary metabolite compositions in every phase [57].

## 3 Biocontrol attributes of entomopathogenic nematodes

Entomopathogenic nematodes showed biocontrol potential against a wide range of insect pests and contributed to the maintenance of insect populations naturally in the environment. In conventional, conservation, and augmentative biological control programmes, EPNs are an emerging and effective choice for managing this insect [58]. More than 200 insect species from various orders within the class Insecta are infected by nematodes, which have a vast host range [59]. EPNs have been successfully used in agricultural systems because of their virulence, motility, quickness, adaptability, and good sense of chemoception [32]. Because of their minimal negative impact on nontarget organisms, using native biocontrol agents to target a particular pest has been adopted. The process of pathogenicity is based on the characteristic interaction between the nematode bacteria and the host insect. It is influenced by insect resistance and by virulence factors of the bacteria and of the nematode acting separately or together to overcome the defence system [54]. EPNs are progressively utilised as biocontrol agents for insect pests (including humoral and cellular defences) [60].After infection, they kill the target host in 24–48 h. They successfully infiltrate and parasitize the larval stages of insect groups that live in soil [23]. The fact that 90 % or more insect pests need to live their entire life cycle in the soil is one of the reasons EPN is used as a bio-agent. Thus, they have been effectively used to combat both folivorous and ground-dwelling insects [31] (Table 2).

**Table 2:** Biocontrol potential of entomopathogenic nematodes against insect pests.

Nematode species	Target insect	Order	Location	Reference
*S. scapterisci*	*Scapteriscus vicinus*	Orthoptera	South America	[225]
*S.carpocapsae* and *H. megidis*	*Otiorhynchus sulcatus*	Lepidoptera	Berkshire, USA	[226]
*S. abbasi*, *S. carpocapsae* and *H. indica*	*A. ipsilon*	Lepidoptera	Bengaluru, India	[227]
*S. monticola*, *S. carpocapsae* and *H*. *bacteriophora*	*S. litura*	Lepidoptera	Korea	[228]
*Heterorhabditis* sp., GAU EPN 16 and *S. riobrave* GAU EPN 3	*Helicoverpa armigera*	Lepidoptera	Gujarat, India	[229]
*S. scarabaei*	Asian garden beetle *Maladera castanea*	Coleoptera	New Brunswick	[230]
*S. riobrave*	*A. ipsilon*	Lepidoptera	Gujarat, India	[231]
*H. mexicana, S. carpocapsae, H. bacteriophora*	*A. ipsilon*	Lepidoptera	USA	[232]
*S. abbasi, S. feltiae, S. carpocapsae, S. glaseri, S. riborave, H. bacteriophora* and *H. indica*	*S. litura*	Lepidoptera	Kerela, India	[233]
*S. carpocapsae* and *S. riobrave*	*Synanthedon exitiosa*	Lepidoptera	Byron, USA	[234]
*H. indica* and *S. glaseri*	*H. armigera*	Lepidoptera	Coimbatore, India	[235]
*H. megidis*	*Otiorhynchus sulcatus*	Lepidoptera	Ireland and Norway	[236]
*S. carpocapsae*	*P. brassicae*	Lepidoptera	Georgia	[237]
*H. zealandica*	*Cydia pomonella*	Lepidoptera	Stellenbosch University, South Africa	[238]
*Steinernema* spp*.* and *Heterorhabditis* spp.	*Synanthedon Pictipes*	Lepidoptera	Columbus, Ohio	[239]
*H. indica*	*Plutella xylostella, H. armigera*, *Leucinodes orbonalis*, *Earias vittella, S. litura, Pieris brassicae, Spilosoma obliqua,* and *Holotrichia consanguinea*,	Lepidoptera and Coleoptera	Indian Agricultural Research Institute, New Delhi, India	[240]
*H. bacteriophora S. feltiae* isolate IRA24 and *S. carpocapsae*	*H. armigera*	Lepidoptera	North-western Iran	[241]
*H. indica, S. feltiae, H. bacteriophora H. Georgiana, S. riobrave* and *S. carpocapsae*	*Corythucha ciliata* and *Stethobaris nemesis*	Hemiptera and Coleoptera	Florida,USA	[242]
*H. bacteriophora*	*A. ipsilon*	Lepidoptera	Jammu and Kashmir, India	[243]
*S. kraussei*	*A. segetum*	Lepidoptera	Turkey	[244]
*S. carpocapsae* and *H. indica*	*S. litura*	Lepidoptera	Bengaluru, India	[245]
*S. carpocapsae*	*Mamestra brassicae*	Lepidoptera	Rumbeke-Beitem, Belgium	[246]
*S.carpocapsae, S. feltiae, H.bacteriophora, H. megidis* and *H. indica*	*Herpetogramma phaeopteralis*	Lepidoptera	Florida, USA	[247]
*S. feltiae, S. carpocapsae, H. bacteriophora*	*Thrips tabaci*	Thysanoptera	India	[248]
*H. bacteriophora*	*P. brassicae*	Lepidoptera	Kashmir, India	[249]
*S. masoodi*	*H. armigera*	Lepidoptera	Varanasi, India	[250]
*S. abbasi, S. masoodi, S. seemae, H. indica* and *H. bacteriophora*	*H. armigera*	Lepidoptera	Tarai region of Uttar Pradesh, India	[251]
*H. bacteriophora* and *S. carpocapsae*	*A. segetum*	Lepidoptera	Iran	[252]
*S. riobrave*	*Helicoverpa zea*	Lepidoptera	Georgia, USA	[253]
*S.carpocapsae, S. feltiae* and *H.bacteriophora*	*Leptinotarsa decemlineata*	Coleoptera	Czech Republic	[254]
*S. carpocapsae*	*S. litura*	Lepidoptera	Udaipur, India	[255]
*Heterorhabditis* sp. (HSG isolate), *Heterorhabditis* sp. (HKM isolate) and *H. bacteriophora* (HRJ isolate)	*H. armigera, S. litura* and *P. xylostella*	Lepidoptera	Himachal Pradesh, India	[256]
*S. abbasi, S. siamkayai* and *H. indica*	*H. armigera*	Lepidoptera	Uttar Pradesh, India	[257]
*H. bacteriophora* and *S. carpocapsae, S. feltiae*	*Thrips tabaci*	Thysanoptera	Giza, Egypt	[258]
*H. indica* and *S. glaseri*	*A. ipsilon*	Lepidoptera	Coimbatore, Tamil Nadu, India	[259]
*H. bacteriophora* and *S. feltiae*	*P. brassicae*	Lepidoptera	Iran	[260]
*S. glaseri* and *H. bacteriophora*	*S. litura*	Lepidoptera	Faisalabad, Pakistan	[261]
*S. glaseri*	Armyworms and bollworms	Lepidoptera	Faisalabad, Pakistan	[262]
*H. bacteriophora* strain FLH-4-H and *H. indica* strain 216-H, *S. carpocapsae* strain E76-S, *S. feltiae* strain E76-S	*A. ipsilon*	Lepidoptera	Cappadocia Region, Central Turkey	[263]
*S. carpocapsae*	*Pieris rapae*	Lepidoptera	Giza, Egypt	[264]
*H. indica, H. bacteriophora* strain HRJ, *Heterorhabditis* sp. HSG, *Heterorhabditis* sp. strain HKM and *Heterorhabditis* sp. strain HSG	*A. segetum*	Lepidoptera	Palampur, Himachal Pradesh, India	[265]
*H. noenieputensis* and *S. yirgalemense*	*Ceratitis capitata*	Diptera	South Africa	[266]
*H. bacteriophora*	*S. litura*, *C. cephalonica*, *Bombyx mori, G. mellonella* and *Brahmina coriacea*	Lepidoptera and Coleoptera	Nauni, Solan, Himachal Pradesh, India	[267]
*H. indica*	*S. litura*	Lepidoptera	Junagadh, India	[268]
*S. carpocapsae* and *H. indica*	*A. segetum*	Lepidoptera	Shimla, Himachal Pradesh, India	[269]
*Heterorhabditis* spp.	*H. armigera*	Lepidoptera	North West Himalayas	[270]
*H. indica*	*H. armigera* and *S. litura*	Lepidoptera	Nagpur, Maharashtra, India	[271]
*H. bacteriophora, H. baujardi, H. indica, H. zealandica, H. noenieputensis* and *S. jeffreyense, S. yirgalemense*	*Holocacista capensis*	Lepidoptera	Western Cape, South Africa	[272]
*S. yirgalemense*	*Frankliniella occidentalis*	Thysanoptera	South Africa	[273]
*S. carpocapsae* and *H. bacteriophora*	*Heliothis virescens*	Lepidoptera	South Africa	[274]
*S. litura* to *H. indica*, *H. bacteriophora*, *S. longicaudum* and *S. carpocapsae*	*S. litura*	Lepidoptera	Daegu, Republic of Korea	[275]
*H. pakistanensis*	*P. brassicae*	Lepidoptera	Srinagar, Jammu and Kashmir, India	[276]
*H. bacteriophora* and *S. glaseri*	*P. brassicae*	Lepidoptera	Faisalabad, Pakistan	[277]
*S. feltiae* and *H. bacteriophora*	*P. brassicae*	Lepidoptera	Himachal Pradesh, India	[278]
*H. bacteriophora* and *S. aciari*	*Odontotermes obesus* and *A. ipsilon*	Blattodea and Lepidoptera	Assam, India	[279]
*H. bacteriophora*	*A. ipsilon*	Lepidoptera	Assam, India	[280]
*H. amazonensis* strain MC01	*H. armigera*	Lepidoptera	Brazil	[281]
EPNs strains *viz.* NBS1, GNAF1 and MAF2	*S. litura*	Lepidoptera	Tamil Nadu, India	[282]
*H. bacteriophora*	*H. armigera, S. litura* and *A. segetum*	Lepidoptera	Himachal Pradesh, India	[283]
*H. bacteriophora* strain S26	*P. brassicae*	Lepidoptera	Himachal Pradesh, India	[35,284]
*H. bacteriophora*	*S. litura*	Lepidoptera	Himachal Pradesh, India	[285]
Five mid-himalyan isolates of *H. bacteriophora*	*S. litura*	Lepidoptera	Himachal Pradesh, India	[286]
*H. bacteriophora*	*H. armigera, S. litura, A. segetum* and *Mythimna separata*	Lepidoptera	Himachal Pradesh, India	[287]
*S. carpocapsae* and *H. bacteriophora*	*Rhynchophorus ferrugineus*	Coleoptera	Pakistan	[288]
*H. indica*	*Spodoptera frugiperda*	Lepidoptera	Maharashtra, India	[289]
*H. bacteriophora*	*S. litura*	Lepidoptera	Himachal Pradesh, India	[290]
*S. carpocapsae*	*P. brassicae*	Lepidoptera	Himachal Pradesh, India	[291]

## 4 Formulations developed from entomopathogenic nematodes

A considerable enhancement has been made in the past few years for the development of nematode-based formulations. In general, an EPN-based formulation contains an active ingredient, i.e., nematodes, a carrier that may include liquids, solids, and gels, as well as cadavers, and an additive that may be adsorbents, absorbents, surfactants, emulsifiers, humectants, thickeners, antimicrobials, dispersants, and also a protector that protects from UV-rays [61]. These components in the formulation usually enhance the EPNs survival and sustain the pathogenicity of the nematodes. Different types of nematode-based formulations are aqueous suspensions, synthetic sponges, wheat-based gluten matrices, gels (alginate), starch matrices, polyacrylamide gels, clay, charcoal, vermiculite, and powder forms, including water-dispersible granules (Table 3).

**Table 3:** Biocontrol potential of entomopathogenic nematodes against insect pests.

Nematode species	Type of formulation	Product name	Target pest	Manufacturers
*S. carpocapsae* *H.bacteriophora*	Polyether-polyurethane moist sponge	NOBUG	Plant borers	The National Research Centre (NRC), Egypt
*H. bacteriophora*	Wettable powder	Cryptonem	*Thaumatotibia leucotreta,* Apples/codling moth, fruit weevil, black vine weevil, sciarids	River Bioscience, South Africa
	Wettable powder	Grubcide	White grubs, Termites,	Sri Biotech, India
	Inert carrier or clay	Larvanem	Curculionidae, *Otiorhynchus*	Biocont Laboratory spol. s r. o., Czech Republic, Europe
	Inert carrier or clay	Nematop	Curculionidae, *Otiorhynchus*	e-nema GmbH, Europe
	Clay powder	Nemagreen	Chafer grubs, billbugs, Japanese beetle, Oriental beetle, Asiatic garden beetle	e-nema GmbH, Germany
	Clay powder	Nematop	White grubs, black vine weevil	e-nema GmbH, Germany
	Wettable powder	OPTINEM H	*Otiorhynchus sulcatus* and other soil beetles	Agrifutur Srl, Italy
	Wettable powder	*H. bacteriophora*	*O. sulcatus, Balaninus nucum, C. splendana, C. fagiglandana, Diabrotica virgifera, virgifera*	Andermatt Biocontrol AG Swiss Confederation, Italy
	Wettable powder	NEMAPACK HB	*O. sulcatus* and other soil beetles	Bioplanet s.c.a., Italy
	Wettable powder	Nematop	*O. sulcatus*	Biogard Divisone CBC (Europe) Srl, Italy
	Wettable powder	Terranem	*Phyllopertha horticola*, *Serica brunnea*, *Hoplia* spp., Aphodius spp	Koppert Italia Srl, Italy
	Wettable powder	Larvanem	*O. sulcatus*	Koppert Italia Srl, Italy
	Wettable powder	Nemax H	*O. sulcatus* and *D. virgifera*	Serbios Srl, Italy
	Wettable powder Clay	B-GREEN	*P. horticola*	Biobest, Poland
	Wettable powder Clay	LARVANEM	*O. sulcatus, P. horticola, Melolontha* spp.*, A. solstitialis*	Koppert, Poland
*H. indica*	Wettable powder	Soldier and Armour	White grubs, Termites, Ash weevil, Sweet potato weevil	Multiplex, Ponalab, and Camson Biotech, India
*H. indica* LPP30 *H. baujardi* LPP7	Infected cadavers	Infected cadavers of *G. mellonella*	*Conotrachelus psidii*	UENF (Universidade Estadual Norte Fluminense) (state university in Brazil
*H. megidis*	Wettable powder Clay	*Heterorhabditis*-System	*O. sulcatus*	Biobest, Poland
	Wettable powder Clay	Nemasys HM	*O. sulcatus*	Syngenta Bioline Becker Underwood, Poland
	Wettable powder Clay	Exhibilinehm	*O. sulcatus*	Syngenta Bioline, Poland
*S. carpocapsae*	Clay powder	Nemastar	*Capnodis tenebrionis*, leatherjackets, mole crickets and black cutworms	e-nema GmbH, Germany
	Wettable powder	OPTIMEN C	*C. pomonella, C. molesta, Euzophera bigella*	Agrifutur Srl, Italy
	Wettable powder	*S. carpocapsae*	*C. pomonella, Agrotis* spp.*, Rhynchophorus ferrugineus, Tipula* spp.*, Gryllotalpa gryllotalpa, Duponchelia fovealis, Paysandisia archon*	Andermatt Biocontrol AG Swiss Confederation, Italy
	Wettable powder	NEMAPACK SC Palme	*R. ferrugineus, P. archon*	Bioplanet s.c.a., Italy
	Wettable powder	NEMAPACK SC	*C. pomonella*	Bioplanet s.c.a., Italy
	Wettable powder	Nemastar	*R. ferrugineus, P. archon, Cydia* spp.*, Synanthedon* spp.*, Spodoptera* spp.*, Agrotis* spp.*, D. fovealis, Amphimallon solstitialis, Rhizotrogus aestivus, C. tenebrionis, G. grillotalpa, Tipula paludosa, T. oleracea*	Biogard Divisone CBC (Europe) Srl, Italy
	Wettable powder clay	Capsanem	Noctuidae, Pyralidae, Tipulidae, Coleoptera and *G. gryllotalpa*	Koppert, Poland
	Wettable powder clay	Exhibitline Sc	*Agrostis* spp., *Tipula* spp.	Syngenta Bioline, Poland
	Wettable powder clay	Nemabact	Western flower thrips, wireworm	OOO Biometodika, Russia
*S. pakistanense*	Sponge	PakNema-1	American, spotted, pink bollworm and root-knot nematodes	NNRC, Pakistan
*S. maqbooli*	Sponge	PakNema-2	Root-knot nematodes	NNRC, Pakistan
*S. bifurcatum*	Sponge	PakNema-3	Pupal stages in soil and termite	NNRC, Pakistan
*S. balochiense*	Sponge	PakNema-4	Fruit fly pupa	NNRC, Pakistan
*S. feltiae* *S. feltiae*	Inert carrier or clay	Entonem	Sciaridae – larvae	Biocont Laboratory spol. s r. o., Czech Republic, Europe
	Inert carrier or clay	Nemaplus	Sciaridae – larvae	e-nema GmbH, Europe
	Inert carrier or clay	*Steinernema*-System	Sciaridae – larvae	Biobest NV, Europe
	Clay powder	Nemaplus	Glasshouse sciarids	e-nema GmbH, Germany
	Clay powder	Nemapom	*Cydia pomonella Synanthedon myopaeformis*	e-nema GmbH, Germany
	Wettable powder	OPTIMEN F	*Cydia pomonella*	Agrifutur Srl (Italy)
	Wettable powder	*S. feltiae*	*Sciaridae, C. pomonella,* thrips	Andermatt Biocontrol AG (Swiss Confederation), Italy
	Wettable powder	NEMAPACK SF	*Sciarids, phorids, Agromyza* spp.*, muscids, nottuids,* *Agrotis* spp.*, Cossus* spp.*, sesids*	Bioplanet s.c.a., Italy
	Wettable powder	Nemaplus	*Cydia* spp.*,Synanthedon* spp.*, Frankliniella occidentalis, Bradysia* spp.*, Acrolepiopsis assectella, Ephrytidae, Scatella stagnalis, Scatellatenui costa, Parahypopta caestrum, Lyriomiza* spp.*, Tuta absoluta*	Biogard Divisone CBC (Europe) Srl Italy
	Wettable powder	Entonem	*Sciarids and thrips*	Koppert Italia Srl, Italy
	Wettable powder	Nemapom	*Cydia* spp.*, Synanthedon* spp. *C. tenebrionis*	Biogard Divisone CBC (Europe) Srl, Italy
	Wettable powder clay	SCIARID	*F. occidentalis, Sciaridae, Diptera*	Koppert, Poland
	Wettable powder clay	Nemasys F	*F. occidentalis, Sciaridae, Diptera*	Syngenta Bioline, Poland
	Wettable powder clay	Exhibitline Sf	*F. occidentalis, T. tabaci*	Syngenta Bioline, Poland
	Wettable powder clay	Entonem	*Thrips tabaci, P. horticola*	Koppert, Poland
	Wettable powder clay	Steinernema- System	*F. occidentalis, Sciaridae, Diptera*	Biobest, Poland
	Wettable powder clay	Entonem F	Western flower thrips, wireworm, *Melolontha chafers*	OOO Biometodika, Russia
*S. kraussei*	Wettable powder clay	Exhibitline Srb and Nemasys L	*O. sulcatus*	Syngenta Bioline, Poland
*S. braziliense*	Sponge	Bio Steinernema	*Sphenophorus levis*	Bio Controle, Brazil

### 4.1 Storage in the form of aqueous suspension

The most common method adapted for EPN storage is the aqueous suspension form. The methods have also been employed for the transportation as well as applications of EPNs [62]. The storage of several species of EPNs at a lower temperature (4–15 °C) increased their survival rates [63]. The storage of *Steinernema* spp. and *Heterorhabditis* spp. at this temperature range has survivorships of 6–12 months and 3–6 months, respectively. However, in several species, low temperatures typically reduce EPN mobility and enzymatic activity, which severely impairs IJ function [64]. Although nematode application is a cost-effective method, the necessity of refrigeration and transportation may enhance their cost [[65], [66], [67]]. The nematode's survival is also influenced by other factors such as nematode concentration, high oxygen requirement, sedimentation, highly reduced temperature ranges for some species, and contamination by microbes [68]. It was reported that the optimum relative humidity was required by *S. feltiae* and *H. bacteriophora* (>90 %) for their survival, while *S. carpocapsae* could survive at lower relative humidity (74 %), as rapid desiccation is the major limiting factor in nematode survival [69]. The nematode's survival also depends on the pH and oxygen concentration. There have been reports that suggest that the increased pH and decreased oxygen concentrations affect the viability of *S. carpocapsae* and Steinernema glaseri (Ssteiner) [70]. Nematode mortality can result from microbial contamination, particularly that caused by fungi, which can lower the quantity of oxygen available and compete with the nematodes [71].

### 4.2 Storage in the form of infected cadaver

The IJs can be stored in the form, of infected cadavers. *Galleria mellonella* (Linnaeus) larvae are the most frequently employed for this purpose due to their excellent qualitative value and high IJ-production rates [72]. Because the cadaver defends the released IJs from damaging biotic and abiotic elements, they have more energy reserves, are better able to spread and infect the host, and can survive longer in the soil [73]. A mean of 14,59,205 and 18,98,512 IJs can be produced per gram of host by employing wax moth larvae [74]. This technique allows small-to medium-sized farmers to use infected insect cadavers for insect pest control [75]. The breaking and rupturing of the insect carcass during its transportation, storage, and shipment is a drawback of employing this formulation approach. This restriction can be addressed, though, by covering the insect cadaver with a protective substance, such as clay or starch [76].

### 4.3 Storage in the form of inert carriers

Nematode storage in the form of an inert carrier is the most suitable method to keep the nematode in small quantities under the refrigerator. The commonly used carriers are in the form of sponge storage and vermiculite. The sponge-based formulation contained a polyether-polyurethane sponge on which nematode suspension was applied at a rate of 500–1000 IJs/cm^2^. The water was also applied to wet the remaining part of the sponge, and the sponge was kept inside the zip-lock plastic bag that was placed under the refrigerator (5–10 °C). The EPN survivorship inside these bags is up to 3 months [65,71]. Another inert carrier was vermiculite, which was a much upgraded form of EPN storage as well as transportation.

In vermiculite, the concentrated nematode suspension is mixed with micronized vermiculite and then kept under the zip-lock plastic bag for storage. The vermiculite was much more stable and easy to apply [68]. The EPN formulation containing EPNs *S. carpocapsae*, *S. feltiae,* and *S*. *longicaudum* (Shen and Wang) upon mixing with humus and vermiculite showed 90 % survivability after 120 days at 5 °C [77]. Recently, *S. feltiae* was stored at different temperature ranges with the use of a polyacrylamide gel and vermiculite mixture. About 80 % viability was recorded in stored nematodes after 241 days at 15 °C, which is reduced to 233 days at 25 °C and about 30 days at 35 °C [78].

### 4.4 Storage in the form of gels

The gel-based formulation of EPNs through encapsulation was first introduced by Kaya and Nelsen [79]. The EPN encapsulations include calcium alginate gel that was prepared for the slower release of nematode juveniles. However, it was not so successful. Polyacrylamide was used by Bedding et al. [80] to contain partially desiccated EPNs, but this storage gel was very difficult to dissolve and has low nematode survivability [68]. Much perfection in EPN storage and viability was achieved by Georgis [81] when he used calcium alginate-based sheets dispersed on plastic screens. The macrogel-based matrix containing mono-glycerides and diglycerides from the vegetable oil was also utilised for the encapsulation of *S. carpocapsae,* which considerably enhanced the nematode viability [82]. The method was further improved with the addition of acrylamide and hydrogenated vegetable oil, which increased the *S. carpocapsae survival rate by* up to 80 % for 35 days [83]. The osmotic treatment was applied to the same material (acrylamide and hydrogenated vegetable oil) before the development of the encapsulated formulation. When the formulation of *S. feltiae* was developed from this material, it showed 99.8 % viability even after six months [62].

The hydrogel, kaolinite, and calcium alginate-based capsule formulations, including those of *H. bacteriophora* and *S. carpocapsae,* showed an EPN viability of 50 % after 40 days [84]. Navon and his co-workers developed calcium alginate-based formulations using EPNs *S. feltiae*, *H. bacteriophora*, *S. carpocapsae,* and *Steinernema riobravae* (Cabanillas, Poinar, and Raulston) and applied them against lepidopteran insect pests such as *Spodoptera littoralis* (Boisduval) and *Helicoverpa armigera* (Hübner) [85]*.* The NemaGel formulation of *Steinernema abbassi* (Elawad, Ahmad, and Reid) can be viable for about 9 months with a survival rate of 89 % in the temperature range of 15 °C–39 °C. However, the nematode stored in alginate gel beads can be escaped out of these beads due to the ultra-softness of the beads [86,87]. This might also happen due to the increased temperature during storage [88].

### 4.5 Storage in the form of clay

The sandwich model introduced by Bedding [89] is used for partial desiccation and the elimination of extra moisture from the body surface of the nematodes. The nematode juveniles were blended with clay and stored in a sandwich form that consisted of two layers of clay, and in-between the nematode juveniles were placed. *S. feltiae*, *Steinernema bibionis* (Bovien), *S. glaseri,* and *Heterorhabditis heliothidis* (Khan, Brooks, and Hirschmann) were used to develop this sandwich formulation that contained hygroscopic attapulgite clay with 8 weeks of viability. Although the formulation was commercialised and sold, it was soon terminated due to poor shelf life, poor storage, poor solubility, clogging during application, and a lower nematode-clay ratio [68].

Several studies indicate that EPNs may also be stored in the infected cadaver [90]. There is much research evidence that the use of an infected cadaver was much superior to the use of an use of an aqueous storage suspension [91]. However, it was found that the infected cadaver's formulation may have several problems in transportation and applications that resulted in a decrease in effectiveness. Ansari and his co-workers coated the infected cadaver with a kaolin-starch mixture and evidenced the maximum nematode viability even after 1 year, which caused 90 % mortality in *Hoplia philanthus* (Fuesslin) [92]. The infected cadaver-based formulations of *S. carpocapsae, S. feltiae,* and *H. bacteriophora* were covered with starch and clay by Lacey et al. [93]. The coated cadavers were again laminated with anti-desiccants such as wood flour foam that showed significant mortality in *Cydia pomonella* (Linnaeus) moth larvae upon its application in mulch and in aqueous suspensions.

### 4.6 Storage in the form of granules

The granular formulations of nematodes in the form of pellets were developed by Capinera and Hibbard [94]. The pellets contained a mixture of multiple components, including corn oil, alfalfa meal, wheat bran, wheat flour, and water. The pellet formulation containing *S. feltiae* was applied against *Melanoplus* spp. in field conditions, resulting in 78.1 % mortality. Connick et al. [95] encapsulated *S. carpocapsae* under a wheat flour-based gluten matrix called “Pesta''. The nematode viability inside this Pesta was quite low (6 weeks), and the risk of contamination by bacteria and fungi was very high. However, the formulation was dried to overcome the contamination issues, but the viability of the nematode was also reduced. Additionally, the dried granules turned into hard pellets that did not dissolve; even a 0.2 % formaldehyde solution was added during the preparation of Pesta to reduce the stress of microbial contamination [96]. The granular formulations developed from diatomaceous earth, amorphous silica, hydroxyethylcellulose, pregelatinized starch, lignosulfonate, starch, pregelled attapulgite clay, and fumed hydrophobic silica that contained *S. carpocapsae*, *Steinernema scapterisci* (Nguyen and Smart), *S. feltiae*, and *S. riobravis* showed 90 % viability when stored for up to 6 weeks. The 19 formulations, including combinations of starches, flours, clays, etc., were tested by Shapiro-Ilan et al. [76] to store *H. bacteriophora.* They found the formulated cadavers were amenable to desiccation, more resistant, and did not stick together.

### 4.7 Storage in the form of activated charcoal

The nematode can be stored in the form of activated charcoal. Yukawa and Pitt [97] mixed the nematodes with activated charcoal powder, which acts as an absorbent material. But the method was costly, difficult to apply, and had poor nematode viability and storage.

### 4.8 Storage in the form of wettable powder

The dispersible granular formulation contained a combination of materials like silica, starches, clays, cellulose, and lignin, which were used to encapsulate nematode juveniles [98]. The recent research tactics focused on the enhancement of viability, storage efficiency, effectiveness, and increased field persistence [99]. The wettable powder formulations have the ability to suspend in water. Nagesh et al. [100] granted a patent on the development of a silicate-based wettable powder mixture that contained IJs of *H. indica* strain NBAII Hi1 and *H. bacteriophora* strain NBAII Hb5. The diatomaceous earth powder (present naturally in the environment in the form of sedimentary rocks) was also utilised for the nematode formulation [101]. Recently, Kagimu and Malan [88] used diatomaceous earth powder for the preparation of EPN formulations that contained *Steinernema yirgalemense* (Nguyen, Tesfamariam, Gozel, Gaugler and Adams), *Steinernema jeffreyense* (Malan, Knoetze and Tiedt), and *H. bacteriophora* with high viability at 14 °C–25 °C temperature ranges. Cortés-Martínez et al. [102] reported longer survivability of *S. glaseri* upon pelletization with diatomaceous earth powder. High efficacy was also recorded when applied against *Phyllophaga vetula* (Horn).

## 5 Applications and future outlook

Generally, the most familiar methodology for EPN application is in the form of aqueous suspension. The nematode juveniles stored in the form of wettable powder, vermiculite, were suspended in water and applied. The juveniles stored in polyether-polyurethane sponges were also squeezed directly and suspended in the water. The gel-based formulations were not suspended in water, so a sodium citrate solution was also added to suspend the alginate gels [81]. Another important aspect was the regular shaking of the suspension during its application, as the suspended nematodes settled at the base of the water. Proper oxygen supply and mixing are required for the consistent distribution of EPNs [68,103]. Shapiro-Ilan et al. [104] suggested the nematode application time be in the early morning and evening, when the temperature is colder, so that the sprayed nematodes do not get desiccated. The nematodes can be applied using mist blowers, hand sprayers, pressure sprayers, and electrostatic sprayers [103].

Nematode has been recognised as an excellent biocontrol agent against a wide range of insect pests. Both forms of insects, above ground and below ground, are highly susceptible to nematode infection. The biocontrol potential of EPNs has been studied not only in the laboratory but also in green house and field conditions, and the nematode-based products (formulations) have been commercialised throughout the world, including agricultural and horticultural practices. However, it is not accepted by the farmers to that extent. So few research needs in the future include: cost reduction in the nematode-based formation as these products are too expensive; easy availability in the market to bring profit to the farmers; production efficiency should be enhanced with longer shelf life and better stability; requirements of the professional market; improved formulation should be developed to increase the viability in extreme temperatures so that farmers can use it easily; and farmers should be educated on the adaptation of EPNs as biocontrol agents.

## 6 Conclusions

This review identified the major concerns about why EPNs should be used for insect pest management. EPNs are ubiquitous in nature and have been successfully utilised as biocontrol agents for the management of insect pests. The nematodes, along with their endosymbiotic bacteria, combine to form a nemato-bacterial complex. This complex is responsible for causing mortality in insect pests within 72 h of infection due to the production of insecticidal compounds. The adaptation of EPNs is an eco-friendly method, economical, and safer for the environment as well as non-target organisms. Moreover, it's a better alternative to synthetic chemical pesticides, as it can be helpful in overcoming pest resistance and resurgence issues. Application of nematode juveniles is a cost-effective method, but the necessity of refrigeration and transportation may enhance their cost. But there are some biotic and abiotic stresses that affect the bio-efficacy of the nematodes and result in poor insect management. The nematode-based formulations are, although easy to apply, difficult to keep for a longer period of time. The attack of microbial contaminants is the limiting factor in the storage of EPNs. Temperature, storage, low oxygen, and transportation also affected the EPN's viability. Other factors that predominantly reduced the usage of nematodes for insect pest management are: the nematode formulations are usually more costly than the chemical-based pesticides; the mass production process is long and the *in vivo* process requires equipment and labour; the insect population increased the costs; the preparation of formulations, storage, and transportation also made formulations expensive; the nematode suspension should be applied in the evening and early morning to avoid sunlight exposure; and the nematode application was followed by irrigation to maintain humidity and efficacy.

## Ethical approval

Not applicable.

## Consent to publish

Not applicable.

## Data availability statement

Not applicable.

## CRediT authorship contribution statement

**Preety Tomar:** Writing – original draft. **Neelam Thakur:** Writing – review & editing. **Sangram Singh:** Writing – review & editing. **Sanjeev Kumar:** Writing – review & editing. **Sarvesh Rustagi:** Writing – review & editing. **Ashutosh Kumar Rai:** Writing – review & editing. **Sheikh Shreaz:** Writing – review & editing. **Neelam Yadav:** Writing – review & editing. **Pankaj Kumar Rai:** Writing – review & editing. **Ajar Nath Yadav:** Writing – review & editing, Conceptualization.

## Declaration of competing interest

The authors declare that they have no known competing financial interests or personal relationships that could have appeared to influence the work reported in this paper.
